# Comparison of diurnal variations, gestational age and gender related differences in fetal heart rate (FHR) parameters between appropriate-for-gestational-age (AGA) and small-for-gestational-age (SGA) fetuses in the home environment

**DOI:** 10.1371/journal.pone.0193908

**Published:** 2018-03-09

**Authors:** Habiba Kapaya, Richard Jacques, Dilly Anumba

**Affiliations:** 1 Department of Oncology and Metabolism, Academic Unit of Reproductive & Developmental Medicine, The University of Sheffield, Sheffield, United Kingdom; 2 Medical Statistics Group, School of Health and Related Research (ScHARR), University of Sheffield, Sheffield, United Kingdom; University of Washington, UNITED STATES

## Abstract

**Objective:**

To assess the influence of gender, time of the day and gestational age on fetal heart rate (FHR) parameters between appropriate-for-gestational-age (AGA) and small-for-gestational age (SGA) fetuses using a portable fetal ECG monitor employed in the home setting.

**Methods:**

We analysed and compared the antenatal FHR data collected in the home setting on 61 healthy pregnant women with singleton pregnancies from 24 weeks gestation. Of the 61 women, 31 had SGA fetuses (estimated fetal weight below the tenth gestational centile) and 30 were pregnant with AGA fetuses. FHR recordings were collected for up to 20 h. Two 90 min intervals were deliberately chosen retrospectively with respect to signal recording quality, one during day-time and one at night-time for comparison.

**Results:**

Overall, success rate of the fetal abdominal ECG in the AGA fetuses was 75.7% compared to 48.6% in the SGA group. Based on randomly selected episodes of heart rate traces where recording quality exceeded 80% we were able to show a marginal difference between day and night-time recordings in AGA vs. SGA fetuses beyond 32 weeks of gestation. A selection bias in terms of covering different representation periods of fetal behavioural states cannot be excluded. In contrast to previous studies, we neither controlled maternal diet and activity nor measured maternal blood hormone and heart rate as all mothers were monitored in the home environment.

**Conclusion:**

Based on clinically unremarkable, but statistically significant differences in the FHR parameters between the AGA and SGA group we suggest that further studies with large sample size are required to assess the clinical value of antenatal fetal ECG monitoring.

## Introduction

Antepartum cardiotocography (CTG) is widely used for the assessment of fetal well-being, although there is no high-quality evidence to indicate that it improves perinatal outcomes [1, 2]. Such lack of evidence may be partly explained by the limited understanding of the influence of gestational age, fetal weight, gender and time of the day on the interpretation of CTG patterns. Nonetheless, some evidence of improved predictive performance has been reported with the use of computer analysis, which is more objective and consistent [3].

Studies have shown changes in CTG parameters with gestation in appropriate-for-gestational-age (AGA) and small-for-gestational-age (SGA) fetuses [3–7]. In addition, some studies suggest differences in fetal heart rate (FHR) patterns between male and female fetuses [1, 8] and between the day or night recordings [9–12]. However, all previous studies were carried out in hospital in clinical settings in which maternal activity is controlled rather than under domestic conditions which are more likely to reflect normal day time maternal activities. Furthermore, there is limited data on the FHR variables as most studies utilised visual rather than computerised system analysis of FHR tracings. Furthermore, to our knowledge, no previous study has compared the influence of gestational age, time of day and fetal gender on FHR patterns between AGA and SGA fetuses concurrently.

The aim of our present study was to explore the effects of diurnal variation, gestational age and gender on computerized CTG in a cohort of healthy pregnant women carrying AGA and SGA fetuses in their home environment using a portable abdominal fetal ECG monitor (Monica Healthcare, Nottingham, UK).

## Material and methods

This work was undertaken to analyze and compare FHR data published separately on normal [6] and SGA fetuses [7] that were monitored in the home environment. Both prospective studies were carried out according to ethics approval from the University and Affiliated Teaching Hospitals Research Ethic Board. The study on small for gestational age fetuses was approved by North-West Preston NRES Research Ethics committee (15/NW/0278) and on appropriate for gestational age fetuses was approved by Research Ethics Committee (REC reference number: 07/Q0108/127). A total of 61 maternal-fetal pairs between 24 and 40 weeks gestation provided the data for this study. Inclusion criteria for both groups were a maternal age of ≥18 years with a singleton pregnancy >24 weeks gestation confirmed by early ultrasound scan.

For the purpose of this study, we used the definition by the Royal College of Obstetricians and Gynaecologists (RCOG) [13] which informs UK clinical practice, based on sonographic estimated fetal weight (EFW) measurement <10^th^ percentile to describe a fetus that has not reached its target weight. Patients were divided into two groups for comparison; fetuses with EFW below the 10^th^ percentile for gestational age (SGA) and fetuses with EFW >10^th^ percentile for gestation (AGA). Exclusion criteria were known fetal malformation, alcohol dependence and any pharmacologically treated co-morbid conditions (e.g. diabetes, hypertension, thyroid diseases, and cardiac problems). None of the participants used medications including β-blockers, central nervous system depressants, tocolytics or steroids which could possibly affect the FHR. Information about each pregnancy, the characteristics of deliveries and newborn outcomes were collected from the medical records.

Women provided informed, voluntary, written consent prior to participation. The fetal ECG monitor (Monica AN24) was attached to the participant’s abdomen by placing five skin electrodes in standardized positions [14]. Participants went home, carried on with day-to-day activities whilst wearing the monitor and removed the monitor after 20 hours had elapsed. They were advised to take off the monitor at any time if they experienced any discomfort. The monitor was either collected from participant’s home by the research team or returned by the participant the following day. Daily activity was to the discretion of the participants. No protocol was followed nor were the times of maternal resting and activity recorded.

Fetal electrophysiological data recorded by the monitor were downloaded later via USB connection. The methodology used for signal extraction and analysis has been described in detail by Cohen et al [15]. In order to investigate changes in FHR pattern between the day and night times, three consecutive 30 min frames (90 min data) with FHR acquisition success rate of >80% were selected randomly during the “day” (7:00–23:00) and “night” (23:00–7:00) periods of the same individual. The FHR parameters studied from the Dawes Redman analysis for this study were:

- Basal FHR: baseline heart rate
- Long-term FHR variation (LTV): overall: minute-by-minute range of pulse intervals
- Short-term FHR variation (STV) overall: sequential epoch-to-epoch variation. Both the LTV overall and the STV overall are measured in milliseconds (ms).
- Accelerations: increase in FHR above the baseline that lasted longer than 15 s and had an amplitude greater than 10 bpm.
- Episodes of high FHR variation (corresponding to active fetal sleep cycles): episodes in which the LTV in at least 5 out of 6 consecutive minutes was equal to or greater than 32 ms.

In contrast to the ultrasound-based CTG monitors, although the fetal ECG monitor (Monica AN24) uses different signals to acquire FHR data, the monitor relies on the same averaging algorithm as introduced by Dawes and Redman [16] to calculate STV and other automated FHR parameters. In all cases, FHR data selected for the analysis fulfilled the Dawes Redman criteria.

### Statistical analysis

Independent sample t-tests were used to compare the characteristics of mothers and babies between the two groups.

Mean differences in day and night time measurements of FHR parameters were calculated within the AGA and the SGA group. To compare differences between groups, linear models were fitted with FHR as the dependent variable and fixed factors for time of measurement and fetus size as independent variables. An interaction term between the two fixed factors was included to test if there was a difference in the day and night time measurements between the two groups. Generalised estimating equations were used to fit the models to allow for repeated measurements on individuals [17]. Differences between groups and time of recording are visually displayed using marginal means from the fitted models.

Mean differences between genders were calculated within the AGA and the SGA group. To compare differences between genders, linear models were fitted with FHR measurement as the dependent variable and fixed factors for gender and fetus size as independent variable. An interaction term between the two fixed factors was included to test if there was a difference in measurements between genders in the normal and the SGA group. Generalised estimating equations were used to fit the models to allow for repeated measurements on individuals.

Linear models were used to examine the relationship between heart rate parameters measured at night and gestational age at time of recording. Models were fitted with FHR as the dependent variable and a fixed factor for fetus size and gestational age covariate as independent variables. An interaction term between the two independent variables was included to allow for different relationships in the normal and the SGA group. Step wise model selection was used to first test for the interaction, the difference between the two groups and finally the linear trend. At each step, non-significant terms were removed.

## Results

A total of 61 maternal-fetal pairs were studied; 30 participants carried AGA and 31 carried SGA fetuses. The baseline characteristics of the participants and their fetuses are given in Table 1. Although, the two groups were broadly comparable in terms of demographics and gestations both at the time of recording and delivery, gender distribution was significantly different between the two groups with more male fetuses in the AGA group and a significantly higher number of females in the SGA group (P = 0.027). As expected, birth weight was significantly lower in the SGA group (P<0.001).

**Table 1:** Summary statistics for maternal and fetal demographic and obstetric characteristics between the AGA and the SGA.

	AGA (N = 30)	SGA (N = 31)	Difference (95% CI)	P-Value
Age (years)	28.9 (5.6)	30.0 (6.1)	-1.1(-4.1, 1.9)	0.456
Body mass index (kg/m^2^)	26.3 (5.7)	25.2 (6.3)	1.1(-2.0, 4.2)	0.479
Gestational age at recording (weeks)	33.8 (3.9)	35.1 (3.2)	-1.3(-3.1, 0.5)	0.158
Gestational age at delivery (weeks)	38.9 (2.4)	39.0 (1.8)	-0.1(-1.1, 1.0)	0.918
Weight of baby (kg)	3.46 (0.55)	2.93 (0.51)	0.53(0.25, 0.80)	<0.001
Sex of babyMaleFemale	19 (63.3%)11 (36.7%)	10 (34.5%)19 (65.5%)	31.1(6.0, 51.2)	0.027

One hundred and twenty-two recordings (61 day and 61 night) made over 90min segments were analysed from 30 AGA and 31 SGA fetuses. All the parameters from both AGA and SGA fetuses were in the normal range at any times of the observations. Exact time of the day and maternal activity during the recordings could not be reconstructed retrospectively.

**Fig 1:**
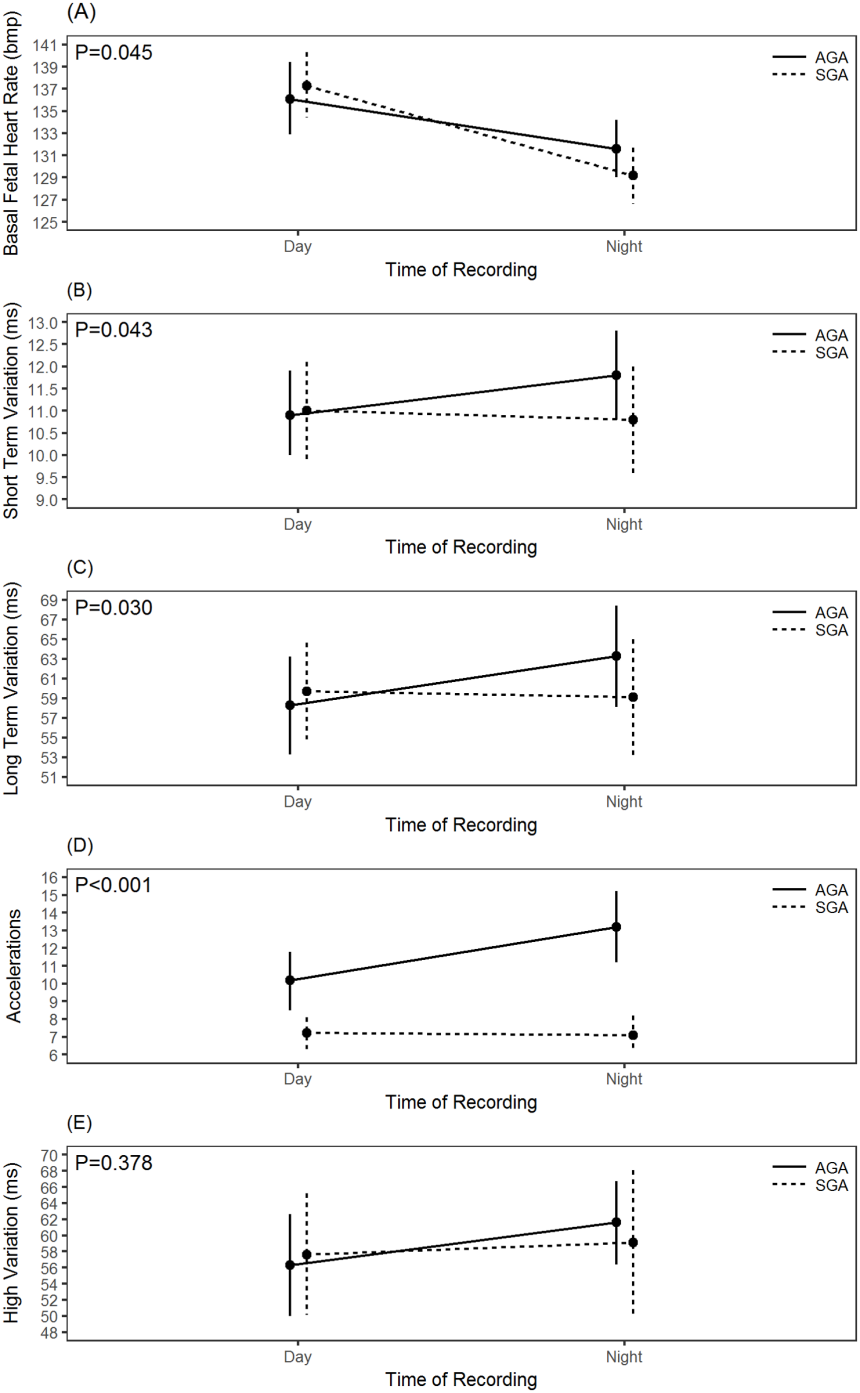
Marginal means plosftc showing differences between day and night recordings of (A) basal fetal heart rate, (B) short term variation, (C) long term variation, (D) accelerations, and (E) high variation.

Table 2 summarizes and compares day: night differences between the two groups. A significant variation in day: night recording was observed in AGA fetuses with a fall in basal FHR and an increase in STV, LTV, number of accelerations and time spent in high variation episode at night time (P ≤ 0.05). The differences in STV, though, are within 1 millisecond of variation. However, SGA fetuses exhibited day: night difference only in basal FHR (P<0.001), whereas STV, LTV, acceleration and time spent in HV remained unchanged (P>0.05). This comparison is further illustrated in Fig 1.

**Table 2:** Comparison of day and night fetal heart rate parameters in AGA and SGA fetuses.

	AGA (N = 30)			SGA (N = 31)			Model Estimate^1^(95% CI)	P-Value
	DayMean(SD)	NightMean(SD)	Difference(95% CI)	DayMean(SD)	NightMean(SD)	Difference(95% CI)		
bFHR (bpm)	136.1(9.2)	131.6(7.4)	4.6(1.5, 7.7)	137.2(8.9)	129.0(6.6)	8.2(1.2, 5.6)	-3.6(-7.1, -0.1)	0.045
STV (ms)	10.9(2.7)	11.8(2.8)	-0.9(-1.7, -0.04)	11.0(2.8)	10.8(3.1)	0.2(-0.5, 1.0)	-1.1(-2.2, -0.04)	0.043
LTV (ms)	58.3(14.0)	63.3(14.8)	-5.0(-8.6, -1.4)	60.1(13.0)	59.4(15.2)	0.7(-3.2, 4.5)	-5.6(-10.8, -0.5)	0.030
Accelerations(number)	10.2(4.7)	13.2(5.6)	-3.0(-4.5, -1.6)	7.3(2.3)	7.3(2.8)	0.1(-1.0, 1.1)	-3.1(-4.8, -1.4)	<0.001
HV (ms)	56.3(18.0)	61.6(14.9)	-5.3(-11.6, 1.0)	58.7(19.1)	60.1(23.0)	-1.5(-7.9, 4.9)	-3.8(-12.2, 4.7)	0.378

^1^The estimate from the model compares differences in measurements from day to night in the AGA and SGA groups.

With respect to gender, even though, the difference in STV between male and female fetuses was higher both in the AGA and the SGA groups than the diurnal variation described above, this observation did not reach statistical significance (Table 3).

**Table 3:** Comparison between AGA and SGA with respect to gender.

	AGA (N = 30)			SGA (N = 29)			Model Estimate^1^(95% CI)	P-Value
	Male Mean(SD)	Female Mean(SD)	Difference(95% CI)	Male Mean(SD)	FemaleMean(SD)	Difference(95% CI)		
bFHR (bpm)	131.3(5.6)	132.0(10.0)	-0.7(-7.7, 6.4)	131.4(5.2)	128.4(7.1)	3.0(-2.2, 8.2)	-2.3(-10.2, 5.7)	0.574
STV (ms)	11.9(2.6)	11.6(2.6)	0.3(-2.1, 2.8)	11.1(2.3)	10.0(2.8)	1.0(-0.9, 3.2)	-1.0(-4.0, 2.1)	0.535
LTV (ms)	64.0(13.6)	62.0(17.2)	2.0(-9.6, 13.7)	61.1(11.7)	55.6(14.8)	5.5(-5.6,16.6)	-3.8(-19.1,11.5)	0.627
Accelerations(number)	13.4(4.4)	12.9(7.7)	0.5(-4.0, 5.0)	7.9(2.6)	6.8 (2.9)	1.1(-1.1, 3.4)	-0.7(-5.7, 4.4)	0.796
HV (ms)	61.3(14.0)	62.1(16.6)	-0.8(-12.3,10.8)	64.2(10.4)	55.2(26.8)	8.9(-9.2,27.1)	-10.2(-28.0, 7.6)	0.260

^1^The estimate from the model compares differences in measurements between male and female fetuses in the AGA and SGA groups

Further analysis of the effect of gestational age on FHR parameters (Table 4) showed no significant difference between AGA and SGA fetuses.

**Table 4:** Correlation between gestational age and fetal heart rate parameters.

	InteractionP-Value^1^	Group Difference^2^		Linear Relationship^3^	
		Estimate (95% CI)	P-Value	Estimate (95% CI)	P-Value
bFHR (bpm)	0.840	1.5 (-1.8, 4.8)	0.375	-0.8 (-1.4, -0.3)	0.003
STV (ms)	0.653	1.0 (-6.1, 2.6)	0.224	-0.03 (-0.3, 0.2)	0.814
LTV (ms)	0.687	4.2 (-3.5, 11.9)	0.286	0.1 (-1.1, 1.3)	0.828
Accelerations (number)	0.516	6.4 (4.0, 8.6)	<0.001	0.3 (-0.1, 0.6)	0.113
HV (ms)	0.700	1.4 (-7.9, 10.8)	0.765	0.1 (-1.5, 1.7)	0.896

^1^ P-Value for interaction between gestational age and group

^2^ Comparison between AGA and SGA groups. A positive value indicates that on average the AGA group have larger measurements

^3^ Change in heart rate parameter per 1 week increase in gestational age

**Fig 2:**
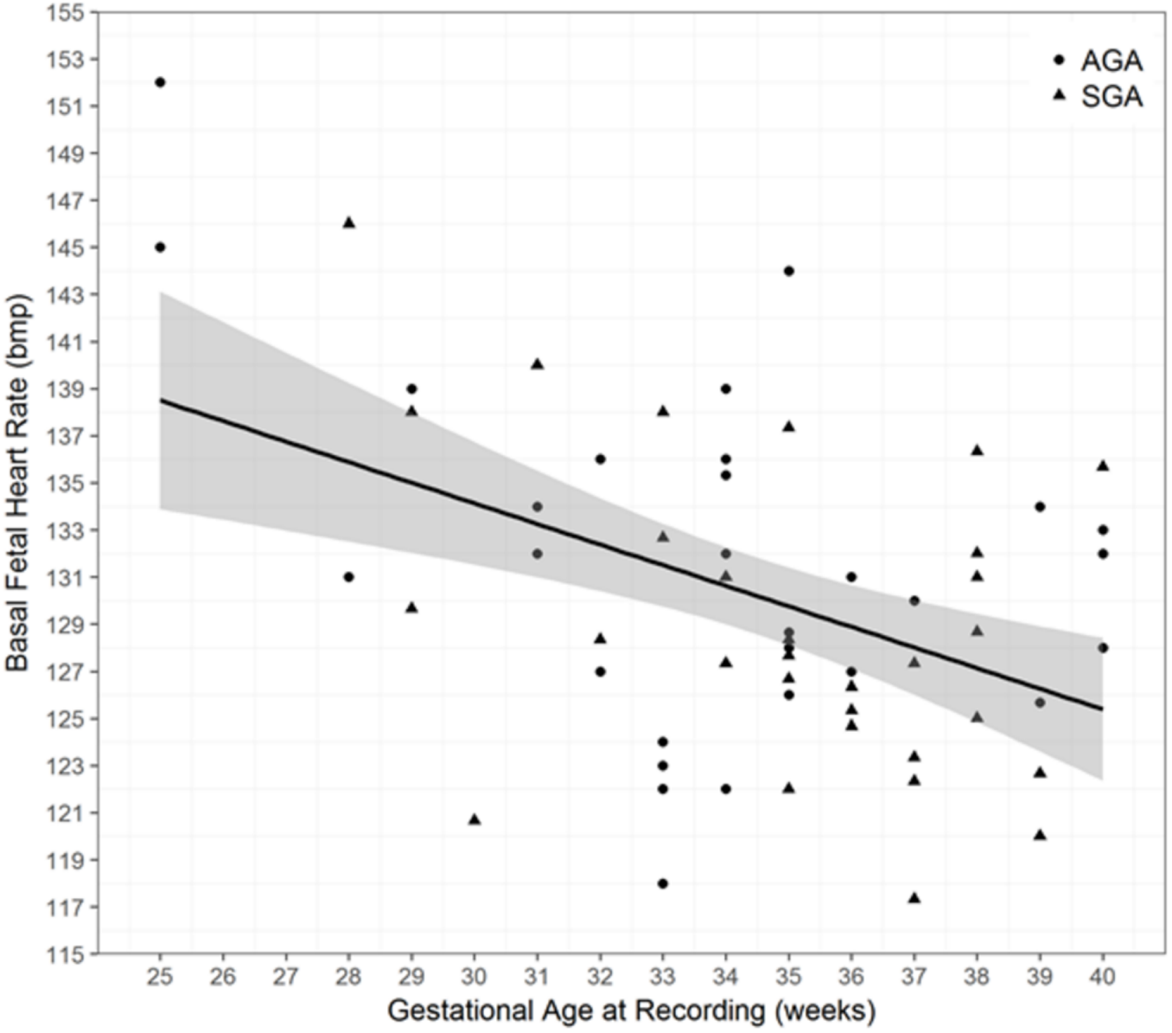
Relationship between gestational age and basal fetal heart rate.

A significant negative correlation between gestational age and basal FHR (Fig 2; P = 0.003) and a weak non-significant positive correlation between LTV, accelerations and time spent in HV and gestational age was observed in both groups (P>0.05).

## Discussion

AGA fetuses exhibited a clinically unremarkable, but in our data statistically significant difference of the Dawes and Redman parameter set between daytime and nighttime recordings. This difference could not be demonstrated in fetuses with known SGA. SGA fetuses showed similar change in FHR pattern with gestational age as AGA fetuses. Gender did not influence CTG interpretation in either group.

This is the first study to compare and report changes in day: night FHR parameters, gestational age and gender related differences in CTG in AGA and SGA fetuses in home environment. Our data was retrieved collecting recordings of the FHR in a home environment over 20 hours. Data quality was as such, that overall 3x30 min both during daytime and night-time met the quality criteria for computerized Dawes and Redman analysis. This is most likely due to the fact, that muscular activity during maternal movement is likely to obscure the fetal ECG from the trans-abdominal recording to a significant proportion.

There are several limitations of this study. There is no consensus on the terminology and diagnostic criteria of fetal growth restriction. The most widely used definition for fetal growth restriction in the United States is an estimated fetal weight (EFW) <10^th^ percentile for gestational age [18]. However, the Royal College of Obstetricians and Gynaecologists (RCOG) considers that fetuses with an EFW or abdominal circumference (AC) <10^th^ percentile, as well as infants with a birthweight <10^th^ percentile are SGA, which is different from fetal growth restriction [13]. SGA includes: 1) babies that have not reached their target growth thorough pathological restriction of the genetic growth potential and possible manifestations of fetal compromise (Doppler abnormalities) and 2) babies that are constitutionally small [19]. Serial ultrasounds can allow identification and differentiation between the two to assess severity and timing of growth restriction. The likelihood of fetal growth restriction is higher in severe SGA cases, defined as an EFW, AC or birthweight <3^rd^ percentile [13]. In the present study, although our SGA cohort did not show any evidence of Doppler changes nor was the EFW below the 3^rd^ percentile at the time of recruitment, our study did not take into account the longitudinal evolution of each fetus, and the existence of a single sonographic finding of EFW measurement <10^th^ centile with normal Doppler studies per fetus was employed for study inclusion.

In the current study, SGA fetuses demonstrated the widely reported phenomenon of maturational change and a similar trend of CTG parameters with increasing gestational age as do the normal fetus. Over the last couple of decades, it has become clear that fetal growth restriction can start early in pregnancy when it is termed as early onset of fetal growth restriction [20]. Figueras and Gratacós [21] and Baschat [22] have reported different behaviors in fetuses with growth restriction before and after 32 weeks gestation [23]. Early onset fetal growth restriction follows a more severe trajectory in terms of neonatal outcome [20] and more pronounced differences in the CTG parameters compared to the late onset growth restricted [24]. In this context, our study was limited and under-representative of SGA fetuses before 32 weeks gestation (only six in the study cohort) which could mean our observations may have occurred by chance or due to selection bias. Due to small sample size we did not undertake separate analysis on SGA<32 week’s gestation. However, when six SGA fetuses under 32 weeks were removed from the analysis, results did not change, implying that our study was representative of less affected SGA that were not truly compromised and therefore remained in utero until later gestational ages. This may explain our observation of a greater overlap in CTG parameters between the normal and SGA fetuses, i.e. a significant decrease in basal FHR and a progressive increase in FHR variation and acceleration with advancing gestation. This is in agreement with the observation made by Soncini et al [25] who found a progressive increase in FHR variability and accelerations with advancing gestational age in intra-uterine-growth restricted (IUGR) fetuses with mild or no Doppler velocimetry abnormalities. However, in more compromised fetuses that showed a greater impairment of placental perfusion, this maturation process of FHR pattern was not observed [25]. A similar observation was made by Amorim-Costa et al [5] who did not find a significant difference in the CTG parameters between the normal and SGA fetuses with birthweight <10th percentiles. However, a pronounced difference was noticed in the SGA group with birthweight <3rd percentiles.

Lack of information on fetal body movements, rest and activity cycles, fetal breathing, maternal activity, diet intake and other physiological conditions that could influence FHR [10, 26, 27], was another limitation of the study. It is therefore not possible to comment on whether the diurnal pattern in the fetus were autonomous or driven by maternal factors.

Another possible limitation of this study was related to the methodology. To assess the day: night change in FHR parameter, we randomly selected the best 90 min FHR data from the day and night recording. This limitation was related to the overall success rate of the recording in the SGA group. The average overall success of acquiring fetal signals in the AGA group was 75.7%, however in the SGA group it was 48.6% which is lower than the success rate reported by Stampalija et al [28]. One of the major problems associated with abdominal fetal ECG monitoring is unpredictable acquisition of fetal signals at any particular point due to widely varying signal: to noise ratio. Contributing factors for poor fetal signals with the abdominal fetal ECG include, maternal activity, environmental electrical noise, gestational age, insulating vernix caseosa and amniotic fluid [14, 29–31]. A lower success rate meant that it was not possible to analyse the whole 20hr recording, therefore we randomly selected the best 90 min data with fetal signals >80% both during the day and night, implying that our study may have suffered selection bias. A healthy fetus cycles between episodes of active and quiet sleep. Active sleep is associated with high variation and quiet sleep is associated with low variation episode. Studies have shown quiet sleep can last between 50–90 min, therefore selection of 90 min intervals guaranteed the analysis of high variation episode [5, 32]. One of the Dawes and Redman criteria for normality is the presence of at least one episode of high variation [33] and this normality criteria was met for all the selected FHR traces.

The diurnal pattern of FHR and its variation exists from 20 to 22 weeks onwards [11]. We observed a significant day: night difference in basal FHR in both AGA and SGA fetuses. However, unlike AGA fetuses, SGA fetuses showed blunting of the diurnal pattern for STV, LTV, accelerations and time spent in HV episode. The reason for the difference in behavior of the SGA fetuses compared to their AGA counterparts is unclear. Our study predominantly comprised of SGA fetuses >32 weeks gestation, implying that gestational age did not affect the altered diurnal pattern observed in SGA group. In addition, our study was designed to exclude other conditions and treatments which could influence diurnal variation of FHR patterns. For instance, studies have shown that in pregnancies complicated by pre-eclampsia, the development of diurnal rhythm in the fetus is hampered [34, 35]. For the purpose of this study, we therefore excluded women with SGA who had pre-eclampsia. In addition, during monitoring, no woman received any medication that could have affected the CTG parameters [36, 37]. However, there were 10 smokers within the SGA group. Although, the information they provided indicates that they did not smoke during the monitoring session, the study relied on the women to provide information on their smoking status rather than making an objective assessment of tobacco exposure. Nicotine consumption has not only been shown to produce greater effect in women but delays the diurnal peak of heart rate pattern [38]. This, may explain why SGA fetuses did not demonstrate the same diurnal pattern in FHR parameters as the normal fetuses’.

To date, only a few studies have evaluated the influence of gender on CTG parameters and these have produced conflicting results. Lange et al [39] suggested that gender does not need to be considered when assessing FHR variability, whereas Fleisher et al [40] and Amorim-Costa et al [1] reported significant gender-related differences in FHR parameters at different gestations. These studies were undertaken on normal fetuses. With regard to SGA pregnancies, there is insufficient data. Kwon et al [8] assessed the influence of gender on FHR between 29 SGA and 385 non-SGA fetuses. Their data suggested no influence of gender in the normal group, but a significant gender-related difference in FHR variability in the SGA group. Our results differ from them, as we did not observe any influence of gender on either of the two groups. This discrepancy can be explained by experimental setting and sample size. We examined the influence of gender on the fetal recordings during the antenatal period whereas, Kwon et al [8] carried out their investigation during labour.

## Conclusion

This study confirms that although SGA fetus with no evidence of Doppler velocimetry abnormalities exhibit similar changes in FHR pattern with gestation as do AGA fetuses, the CTG pattern seems to be differing between the two groups when the time of day is taken into consideration. Nonetheless, all our observations are in the range of normal findings. We did not follow up on perinatal outcome, nor we analysed beat-to-beat information on the acquired FHR data, implying that may be the underlying difference in the STV between the AGA and the SGA were masked by the use of averaged FHR data provided by the Monica AN24 leading to the loss in temporal resolution. We suggest that further studies should be undertaken to explore the value of true beat-to-beat information on FHR variability by employing Monica AN24 before its clinical utility and more widespread adoption can be ascertained.

## Supporting information

S1 Fig Data file Normal_Small babies. (XLSX)

## References

[1] Amorim-Costa C, Cruz J, Ayres-de-Campos D, Bernardes J: Gender-specific reference charts for cardiotocographic parameters throughout normal pregnancy: a retrospective cross-sectional study of 9701 fetuses. *Eur J Obstet Gynecol Reprod Biol* 2016, 199:102–107. doi: 10.1016/j.ejogrb.2016.01.036 26921476 10.1016/j.ejogrb.2016.01.036

[2] Grivell RM, Alfirevic Z, Gyte GM, Devane D: Antenatal cardiotocography for fetal assessment. *Cochrane Database Syst Rev* 2012, 12:CD007863 doi: 10.1002/14651858.CD007863.pub3 23235650 10.1002/14651858.CD007863.pub3

[3] Serra V, Bellver J, Moulden M, Redman CW: Computerized analysis of normal fetal heart rate pattern throughout gestation. *Ultrasound Obstet Gynecol* 2009, 34(1):74–79. doi: 10.1002/uog.6365 19489020 10.1002/uog.6365

[4] Yanagihara T, Hata T: Comparison of late-second-trimester nonstress test characteristics between small for gestational age and appropriate for gestational age infants. *Obstet Gynecol* 1999, 94(6):921–924. 10576176 10.1016/s0029-7844(99)00427-5

[5] Amorim-Costa C, de Campos DA, Bernardes J: Cardiotocographic parameters in small-for-gestational-age fetuses: How do they vary from normal at different gestational ages? A study of 11687 fetuses from 25 to 40 weeks of pregnancy. *J Obstet Gynaecol Res* 2017, 43(3):476–485. doi: 10.1111/jog.13235 28165176 10.1111/jog.13235

[6] Kapaya H, Broughton Pipkin F, Hayes-Gill B, Loughna PV: Circadian changes and sex-related differences in fetal heart rate parameters. *Matern Health Neonatol Perinatol* 2016, 2(1):9 doi: 10.1186/s40748-016-0037-6 27595008 10.1186/s40748-016-0037-6 PMC5010766

[7] K H, D ER, A D: Do small for gestational age foetuses' exhibit circadian changes in fetal heart rate parameters as do appropriate for gestational age foetuses? *Edorium J Matern Child Health* 2017, 2:1–8.

[8] Kwon JY, Park IY, Lim J, Shin JC: Changes in spectral power of fetal heart rate variability in small-for-gestational-age fetuses are associated with fetal sex. *Early Hum Dev* 2014, 90(1):9–13. doi: 10.1016/j.earlhumdev.2013.11.005 24332839 10.1016/j.earlhumdev.2013.11.005

[9] Babazadeh R, Abdali K, Lotfalizadeh M, Tabatabaie HR, Kaviani M: Diurnal nonstress test variations in the human fetus at risk. *Int J Gynaecol Obstet* 2005, 90(3):189–192. doi: 10.1016/j.ijgo.2005.05.011 16043174 10.1016/j.ijgo.2005.05.011

[10] Lunshof S, Boer K, Wolf H, van Hoffen G, Bayram N, Mirmiran M: Fetal and maternal diurnal rhythms during the third trimester of normal pregnancy: outcomes of computerized analysis of continuous twenty-four-hour fetal heart rate recordings. *Am J Obstet Gynecol* 1998, 178(2):247–254. 9500482 10.1016/s0002-9378(98)80008-2

[11] de Vries JI, Visser GH, Mulder EJ, Prechtl HF: Diurnal and other variations in fetal movement and heart rate patterns at 20–22 weeks. *Early Hum Dev* 1987, 15(6):333–348. 3436277 10.1016/0378-3782(87)90029-6

[12] Visser GH, Goodman JD, Levine DH, Dawes GS: Diurnal and other cyclic variations in human fetal heart rate near term. *Am J Obstet Gynecol* 1982, 142(5):535–544. 7199260 10.1016/0002-9378(82)90757-8

[13] Royal College of Obstetricians and Gynaecologists. The investigation and management of the small-for-gestational-age fetus (guideline no. 31). London: Royal College of Obstetricians and Gynaecologists; 2002. In.

[14] Graatsma EM, Jacod BC, van Egmond LA, Mulder EJ, Visser GH: Fetal electrocardiography: feasibility of long-term fetal heart rate recordings. *BJOG* 2009, 116(2):334–337; discussion 337–338. doi: 10.1111/j.1471-0528.2008.01951.x 19076966 10.1111/j.1471-0528.2008.01951.x

[15] Cohen WR, Ommani S, Hassan S, Mirza FG, Solomon M, Brown R, Schifrin BS, Himsworth JM, Hayes-Gill BR: Accuracy and reliability of fetal heart rate monitoring using maternal abdominal surface electrodes. *Acta Obstet Gynecol Scand* 2012, 91(11):1306–1313. doi: 10.1111/j.1600-0412.2012.01533.x 22924738 10.1111/j.1600-0412.2012.01533.x

[16] Seliger G, Petroff D, Seeger S, Hoyer D, Tchirikov M, Schneider U: Diurnal variations of short-term variation and the impact of multiple recordings on measurement accuracy. *J Perinatol* 2017, 37(3):231–235. doi: 10.1038/jp.2016.202 27831546 10.1038/jp.2016.202

[17] Burton P, Gurrin L, Sly P. Tutorial in Biostatistics. Extending the simple linear regression model to account for correlated responses: an introduction to generalized estimating equations and multi-level mixed modelling. Statistics in Medicine 1998, 17:1261–1291. 9670414 10.1002/(sici)1097-0258(19980615)17:11<1261::aid-sim846>3.0.co;2-z

[18] American College of Obstetricians and Gynecologists. Intrauterine growth restriction. Washington, DC: American College of Obstetricians and Gynecologists; 2000. In.

[19] Grivell RM, Wong L, Bhatia V: Regimens of fetal surveillance for impaired fetal growth. *Cochrane Database Syst Rev* 2012(6):CD007113 doi: 10.1002/14651858.CD007113.pub3 22696366 10.1002/14651858.CD007113.pub3 PMC6465035

[20] Nawathe A, Lees C: Early onset fetal growth restriction. *Best Pract Res Clin Obstet Gynaecol* 2017, 38:24–37. doi: 10.1016/j.bpobgyn.2016.08.005 27693119 10.1016/j.bpobgyn.2016.08.005

[21] Figueras F, Gratacos E: Stage-based approach to the management of fetal growth restriction. *Prenat Diagn* 2014, 34(7):655–659. doi: 10.1002/pd.4412 24839087 10.1002/pd.4412

[22] Baschat AA: Neurodevelopment following fetal growth restriction and its relationship with antepartum parameters of placental dysfunction. *Ultrasound Obstet Gynecol* 2011, 37(5):501–514. doi: 10.1002/uog.9008 21520312 10.1002/uog.9008

[23] Nardozza LM, Caetano AC, Zamarian AC, Mazzola JB, Silva CP, Marçal VM, Lobo TF, Peixoto AB, Araujo E Júnior: Fetal growth restriction: current knowledge. *Arch Gynecol Obstet* 2017, 295(5):1061–1077. doi: 10.1007/s00404-017-4341-9 28285426 10.1007/s00404-017-4341-9

[24] Amorim-Costa C, Gaio AR, Ayres-de-Campos D, Bernardes J: Longitudinal changes of cardiotocographic parameters throughout pregnancy: a prospective cohort study comparing small-for-gestational-age and normal fetuses from 24 to 40 weeks. *J Perinat Med* 2017, 45(4):493–501. doi: 10.1515/jpm-2016-0065 27474837 10.1515/jpm-2016-0065

[25] Soncini E, Ronzoni E, Macovei D, Grignaffini A: Integrated monitoring of fetal growth restriction by computerized cardiotocography and Doppler flow velocimetry. *Eur J Obstet Gynecol Reprod Biol* 2006, 128(1–2):222–230. doi: 10.1016/j.ejogrb.2006.01.001 16431011 10.1016/j.ejogrb.2006.01.001

[26] Ozkaya E, Baser E, Cinar M, Korkmaz V, Kucukozkan T: Does diurnal rhythm have an impact on fetal biophysical profile? *J Matern Fetal Neonatal Med* 2012, 25(4):335–338. doi: 10.3109/14767058.2011.576721 21696335 10.3109/14767058.2011.576721

[27] Marzbanrad F, Kimura Y, Palaniswami M, Khandoker AH: Quantifying the Interactions between Maternal and Fetal Heart Rates by Transfer Entropy. *PLoS One* 2015, 10(12):e0145672 doi: 10.1371/journal.pone.0145672 26701122 10.1371/journal.pone.0145672 PMC4689348

[28] Stampalija T, Casati D, Montico M, Sassi R, Rivolta MW, Maggi V, Bauer A, Ferrazzi E: Parameters influence on acceleration and deceleration capacity based on trans-abdominal ECG in early fetal growth restriction at different gestational age epochs. *Eur J Obstet Gynecol Reprod Biol* 2015, 188:104–112. doi: 10.1016/j.ejogrb.2015.03.003 25801726 10.1016/j.ejogrb.2015.03.003

[29] Hasan MA, Reaz MB, Ibrahimy MI, Hussain MS, Uddin J: Detection and Processing Techniques of FECG Signal for Fetal Monitoring. *Biol Proced Online* 2009, 11:263–295. doi: 10.1007/s12575-009-9006-z 19495912 10.1007/s12575-009-9006-z PMC3055800

[30] Stinstra JG, Peters MJ: The influence of fetoabdominal tissues on fetal ECGs and MCGs. *Arch Physiol Biochem* 2002, 110(3):165–176. doi: 10.1076/apab.110.3.165.8293 12221516 10.1076/apab.110.3.165.8293

[31] Sameni R, Clifford GD: A Review of Fetal ECG Signal Processing; Issues and Promising Directions. *Open Pacing Electrophysiol Ther J* 2010, 3:4–20. doi: 10.2174/1876536X01003010004 21614148 10.2174/1876536X01003010004 PMC3100207

[32] Muro M, Shono H, Kohara M, Ito Y, Uchiyama A, Sugimori H: Diurnal variations in resting-active cycles in full-term fetal heart rate changes. *Early Hum Dev* 1996, 44(1):51–58. 8821895 10.1016/0378-3782(95)01691-0

[33] Pardey J, Moulden M, Redman CW: A computer system for the numerical analysis of nonstress tests. *Am J Obstet Gynecol* 2002, 186(5):1095–1103. 12015543 10.1067/mob.2002.122447

[34] Koenen SV, Franx A, Mulder EJ, Bruinse HW, Visser GH: Fetal and maternal cardiovascular diurnal rhythms in pregnancies complicated by pre-eclampsia and intrauterine growth restriction. *J Matern Fetal Neonatal Med* 2002, 11(5):313–320. doi: 10.1080/jmf.11.5.313.320 12389672 10.1080/jmf.11.5.313.320

[35] Ditisheim AJ, Dibner C, Philippe J, Pechère-Bertschi A: Biological rhythms and preeclampsia. *Front Endocrinol (Lausanne)* 2013, 4:47. 23579266 10.3389/fendo.2013.00047 PMC3619120

[36] Piazze J, Dillon KC, Albana C: Full-term-pregnancy effects of antenatal betamethasone administration on short-term variation as assessed by computerized cardiotocography. *J Prenat Med* 2012, 6(2):18–21. 22905307 PMC3421949

[37] Nensi A, De Silva DA, von Dadelszen P, Sawchuck D, Synnes AR, Crane J, Magee LA: Effect of magnesium sulphate on fetal heart rate parameters: a systematic review. *J Obstet Gynaecol Can* 2014, 36(12):1055–1064. doi: 10.1016/S1701-2163(15)30382-0 25668040 10.1016/S1701-2163(15)30382-0

[38] Adan A, Sánchez-Turet M: Influence of smoking and gender on diurnal variations of heart rate reactivity in humans. *Neurosci Lett* 2001, 297(2):109–112. 11121882 10.1016/s0304-3940(00)01687-6

[39] Lange S, Van Leeuwen P, Geue D, Hatzmann W, Grönemeyer D: Influence of gestational age, heart rate, gender and time of day on fetal heart rate variability. *Med Biol Eng Comput* 2005, 43(4):481–486. 16255430 10.1007/BF02344729

[40] Fleisher LA, Dipietro JA, Johnson TR, Pincus S: Complementary and non-coincident increases in heart rate variability and irregularity during fetal development. *Clin Sci (Lond)* 1997, 92(4):345–349. 9176032 10.1042/cs0920345

